# Survival benefit and dynamics of CD8^+^ T cells and tumor-associated macrophages in neoadjuvant immunochemotherapy *vs*. chemotherapy for locally advanced esophageal squamous cell carcinoma: an IPTW-adjusted real-world study

**DOI:** 10.3389/fimmu.2026.1788756

**Published:** 2026-03-26

**Authors:** Yi Gan, Yue Li, Li Fang, Yuejun Chen, Desong Yang

**Affiliations:** 1 Department of Thoracic Surgery, Hunan Cancer Hospital/The Affiliated Cancer Hospital of Xiangya School of Medicine, Central South University, Changsha, Hunan, China; 2 Department of Radiotherapy, Hunan Cancer Hospital/The Affiliated Cancer Hospital of Xiangya School of Medicine, Central South University, Changsha, Hunan, China

**Keywords:** locally advanced esophageal squamous cell carcinoma, neoadjuvant immunochemotherapy, pathological complete response, real-world study, tumor microenvironment

## Abstract

**Background:**

Neoadjuvant immunochemotherapy (nICT) has emerged as a promising strategy for patients with locally advanced esophageal squamous cell carcinoma (LA-ESCC), yet real-world evidence comparing its efficacy and safety with conventional neoadjuvant chemotherapy (nCT) remains scarce. Furthermore, the underlying immune microenvironment dynamics associated with these clinical benefits have not been fully elucidated.

**Method:**

We conducted a retrospective cohort study of patients with resectable LA-ESCC who received either nICT or nCT followed by curative esophagectomy. We used inverse probability of treatment weighting (IPTW) to minimize selection bias. The primary endpoints were overall survival (OS) and disease-free survival (DFS). In addition, we analyzed recurrence patterns and performed multiplex immunohistochemistry (mIHC) to characterize the immune microenvironment associated with treatment response.

**Results:**

A total of 224 patients received nICT and 80 received nCT. After IPTW adjustment, OS in the nICT group was significantly better than in the nCT group (3-year OS: 77.68% *vs*. 67.49%; HR = 0.61, 95% CI: 0.37–0.98, P = 0.04). Notably, adjusted DFS did not differ significantly (P = 0.96). This discrepancy was driven by distinct recurrence patterns: distant organ metastasis was significantly lower in the nICT group (10.71% *vs*. 26.25%, P < 0.01). In exploratory analyses, nICT responders exhibited increased CD8^+^ T-cell infiltration and reduced M2-like macrophages (CD68^+^HLA-DR^-^), suggesting a shift from an immunosuppressive to an immune-activated microenvironment.

**Conclusion:**

nICT provided a significant survival advantage over nCT, primarily by inhibiting distant metastasis rather than preventing local recurrence. This benefit may be attributed to immune microenvironment dynamics, specifically characterized by T-cell activation and macrophage modulation.

## Introduction

Esophageal squamous cell carcinoma (ESCC) remains a major global health challenge, particularly in China, where it is one of the leading causes of cancer-related deaths (1, 2). For patients with locally advanced esophageal squamous cell carcinoma (LA-ESCC), neoadjuvant chemotherapy (nCT) combined with radical esophagectomy has been the standard treatment. Although neoadjuvant chemotherapy can improve tumor staging compared to surgery alone, the long-term efficacy remains unsatisfactory. A considerable number of patients still experience local or distant recurrence, suggesting that chemotherapy alone may be insufficient to eradicate micrometastases in many cases (3–6).

The treatment landscape has recently shifted with the introduction of immune checkpoint inhibitors (ICIs). In several clinical trials, ICIs combined with chemotherapy (nICT) have shown higher pathological complete response (pCR) rates compared to historical control groups (7–12). Encouraged by these findings, many centers have adopted nICT in routine practice. However, the high-quality evidence supporting this shift primarily comes from clinical trials with strict inclusion criteria, which may not fully represent the general patient population.

In real-world settings, comparative data between nICT and nCT are often limited by selection bias, making it difficult to accurately assess their true survival benefits. Furthermore, while the short-term advantages of nICT, such as pCR, have been well-established, its impact on long-term recurrence patterns, particularly whether it is more effective at preventing distant metastasis than local recurrence, remains unclear. Moreover, the biological basis of these clinical benefits still needs to be fully elucidated. Understanding the dynamics of the tumor immune microenvironment (TME) following nICT is crucial for identifying which patients are most likely to benefit.

To address these issues, we conducted a real-world cohort study comparing the efficacy of nICT versus nCT in patients with resectable LA-ESCC. To minimize selection bias in survival analysis while accounting for the substantial group size asymmetry inherent in our real-world cohort, we employed inverse probability of treatment weighting (IPTW). In addition, we performed exploratory multiplex immunohistochemistry (mIHC) analysis to characterize post-treatment changes in the immune microenvironment, aiming to provide translational insights into the immune dynamics associated with clinical responses.

## Materials and methods

This retrospective study included patients with LA-ESCC who underwent neoadjuvant therapy followed by curative esophagectomy at Hunan Cancer Hospital/The Affiliated Cancer Hospital of Xiangya School of Medicine, Central South University, between November 2019 and June 2024. The study was conducted in accordance with the Declaration of Helsinki and approved by the Ethics Committee of Hunan Cancer Hospital (No. 2025605). Written informed consent was obtained from all patients.

The inclusion criteria were as follows: (1) age ≥18 years; (2) histologically confirmed, surgically resectable ESCC (13); (3) receipt of neoadjuvant chemotherapy with or without immune checkpoint inhibitors before surgery; (4) Eastern Cooperative Oncology Group (ECOG) performance status score of 0 or 1 (14); (5) complete clinical and pathological data available; (6) no history of prior malignancies or previous cancer treatments. Patients not meeting the above criteria were excluded.

### Data collection

Demographic and clinical variables were collected, including age, sex, smoking history, tumor histopathology, tumor location, clinical TNM staging, specific neoadjuvant regimens and cycle numbers and final postoperative pathological findings.

### Treatment

All patients received platinum-based neoadjuvant chemotherapy, either alone (nCT) or combined with immune checkpoint inhibitors (nICT). Treatment response and surgical eligibility were evaluated before surgery. Radical minimally invasive esophagectomy with lymphadenectomy was performed 3–6 weeks after the final neoadjuvant cycle (15).

### Endpoints and definitions

The primary endpoint was OS, defined as the time from initiation of neoadjuvant therapy to death from any cause or the last follow-up. Secondary endpoints included pathological complete response (pCR), DFS and patterns of recurrence. DFS was measured from the date of surgery to the first documented recurrence, death, or last follow-up. Pathological response was evaluated by experienced pathologists based on the percentage of residual viable tumor cells in the resected specimen. pCR was defined as the complete absence of viable tumor cells (16–18).

### Perioperative safety assessment

The patient’s overall condition was monitored after each cycle of neoadjuvant therapy. Treatment-related adverse events were documented and graded using the Common Terminology Criteria for Adverse Events (CTCAE, version 5.0). Postoperative complications were evaluated according to the Clavien–Dindo classification, with those of grade 3 or higher defined as major complications (19).

### Recurrence pattern classification

Patterns of first recurrence were analyzed in both the nCT and nICT groups. Recurrence was classified as local tumor recurrence, regional lymph node recurrence (cervical, mediastinal, or upper abdominal nodes), distant organ or soft tissue metastasis, and distant lymph node metastasis, according to AJCC TNM and NCCN guidelines (20, 21).

### Multiplex immunohistochemistry

We performed an exploratory immunoprofiling analysis on 40 patients who received nICT and divided them into two groups based on postoperative pathological results: pathological complete response (pCR, n = 20) and non-pCR (n = 20). Paired pre- and post-treatment formalin-fixed paraffin-embedded (FFPE) tissue sections were stained using multiplex immunohistochemistry (mIHC) under standardized protocols. The antibody panel included antibodies against CD8, CD68, HLA-DR, CD56, PD-L1, Pan-CK and DAPI. Importantly, appropriate staining controls were utilized to verify staining specificity, including negative controls (omitting primary antibodies), isotype-matched controls during assay optimization, and single-plex staining validation prior to multiplex panel assembly.

Quantitative image analysis was performed using QuPath (v0.6) software. Nuclear counterstaining with DAPI was utilized for robust automated single-cell segmentation. Prior to quantitative analysis, stringent quality control procedures were applied to manually exclude low-quality regions, such as tissue folds, necrosis, and out-of-focus areas, to minimize technical artifacts and background noise. We quantitatively analyzed the densities of CD8^+^ T cells, M1-like macrophages (CD68^+^HLA-DR^+^), M2-like macrophages (CD68^+^HLA-DR^-^), and CD56^+^ NK cells in the intratumoral and stromal regions. To differentiate between intratumoral and stromal regions within the same tissue section, we performed computational tissue segmentation based on Pan-CK immunofluorescence. Pan-CK (an epithelial marker) was utilized to identify the epithelial/tumor regions: Pan-CK-positive tumor nests were digitally defined as the tumor parenchyma, while adjacent Pan-CK-negative regions in the tissue section were classified as the tumor stroma.

In addition, PD-L1 expression was evaluated using standard clinical immunohistochemistry (IHC) on pre-treatment biopsy specimens and scored by the combined positive score (CPS).

### Statistical analysis

Data analysis was performed using R software (v4.4.3). Baseline comparisons were conducted using t-tests, Mann-Whitney U tests, or chi-square/Fisher’s exact tests as appropriate. To mitigate selection bias, we used inverse probability of treatment weighting (IPTW) to assess covariate balance, with a standardized mean difference (SMD) < 0.10 indicating optimal balance. Survival outcomes were estimated using the Kaplan-Meier method. To determine the hazard ratios (HRs) for OS and DFS, univariate and IPTW-adjusted multivariate Cox proportional hazards regression models with robust standard errors were constructed. Subgroup analyses were performed using unadjusted Cox models. All tests were two-sided (P < 0.05).

## Results

### Patient characteristics

**Figure 1:**
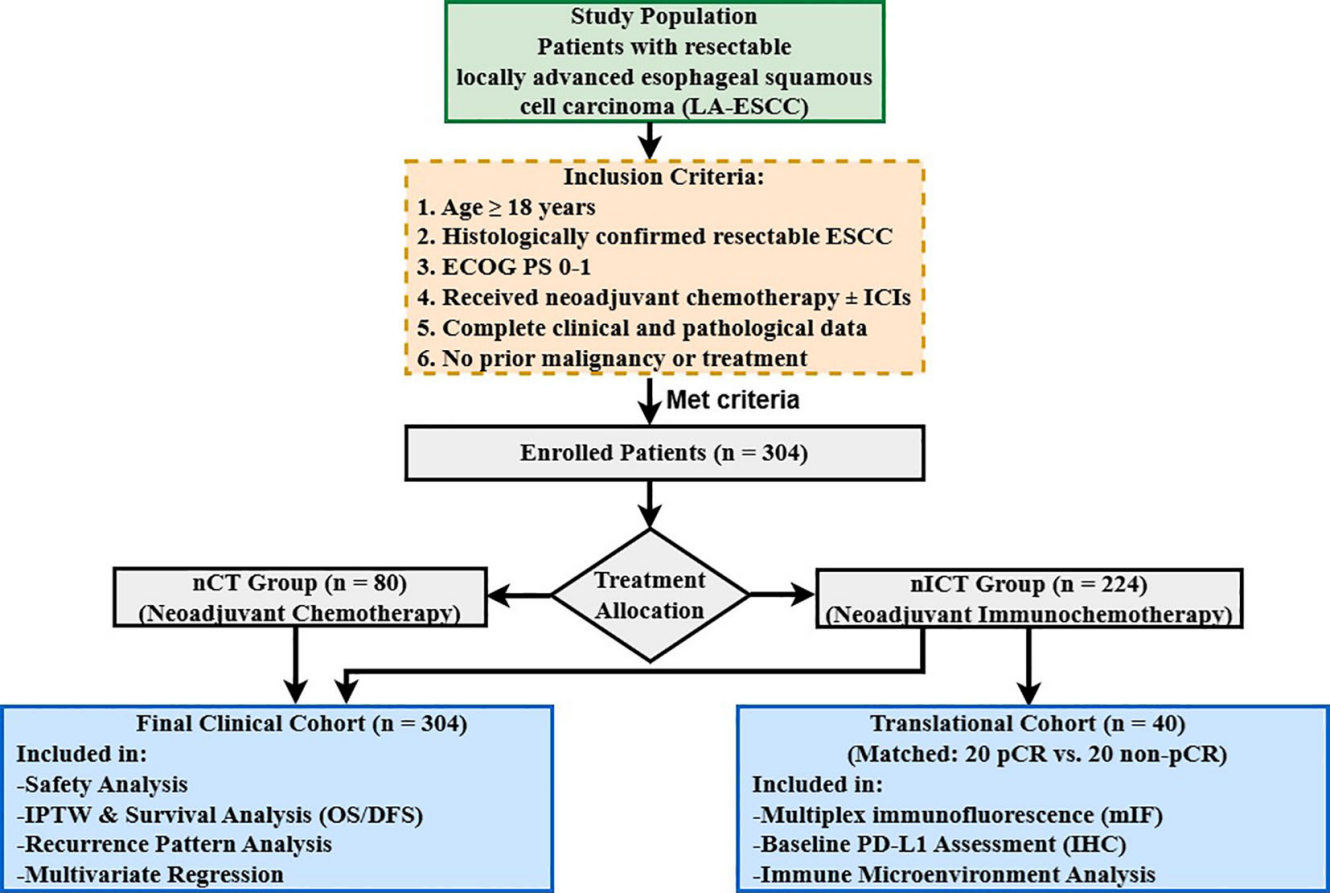
Flowchart of patient enrollment and study design.

A total of 304 patients met the inclusion criteria, including 224 in the nICT group and 80 in the nCT group. The flow chart of this study is shown in **Figure 1**. All patients underwent radical esophagectomy after receiving neoadjuvant therapy. The baseline characteristics of the two groups were generally balanced (P > 0.05), except for the number of neoadjuvant therapy cycles (P < 0.001). To address this imbalance, we performed IPTW adjustment. After adjustment, all covariates achieved optimal balance, with standardized mean differences (SMD) for all variables below 0.10 (**Table 1**).

**Table 1:** Baseline characteristics of patients in the original and weighted cohorts.

Characteristic	Original Cohort (Unweighted)			Weighted Cohort (IPTW)		
	nCT (n=80)	nICT (n=224)	P-value	nCT (ESS = 58.35)	nICT (ESS = 198.50)	SMD
Age (years)*	61.00 ± 7.62	59.60 ± 7.09	0.15	60.28	59.92	0.05
Sex, n (%)			0.88			< 0.01
Male	75 (93.75)	207 (92.41)		93.01	93.00	
Female	5 (6.25)	17 (7.59)		6.99	7.00	
Smoking status, n (%)			0.28			0.02
Never	11 (13.75)	45 (20.09)		19.74	17.85	
Former or current	69 (86.25)	179 (79.91)		80.26	82.15	
Alcohol consumption, n (%)			0.79			< 0.01
Never	19 (23.75)	48 (21.43)		20.75	21.01	
Former or current	61 (76.25)	176 (78.57)		79.25	78.99	
Tumor location, n (%)			0.54			0.01
Upper segment	3 (3.75)	13 (5.80)		6.39	5.35	
Middle segment	33 (41.25)	104 (46.43)		43.11	44.32	
Lower segment	44 (55.00)	107 (47.77)		50.51	50.34	
cT, n (%)			0.22			0.02
2	10 (12.50)	15 (6.70)		9.62	7.40	
3	69 (86.25)	207 (92.41)		89.36	91.63	
4	1 (1.25)	2 (0.89)		1.02	0.98	
cN, n (%)			0.79			0.05
0	20 (25.00)	47 (20.98)		24.58	21.12	
1	27 (33.75)	80 (35.71)		30.86	36.35	
2	32 (40.00)	90 (40.18)		43.54	39.94	
3	1 (1.25)	7 (3.13)		1.02	2.58	
cStage, n (%)			0.64			0.02
2	19 (23.75)	52 (23.21)		24.19	22.92	
3	60 (75.00)	163 (72.77)		74.79	73.66	
4	1 (1.25)	9 (4.02)		1.02	3.42	
ECOG PS, n (%)			1.00			0.01
0	75 (93.75)	210 (93.75)		94.70	93.81	
1	5 (6.25)	14 (6.25)		5.30	6.19	
Number of neoadjuvant therapy cycles, n (%)			< 0.01			0.07
≤ 2	72 (90.00)	136 (60.71)		75.56	68.36	
> 2	8 (10.00)	88 (39.28)		24.44	31.64	

*mean ± standard deviation; cT, clinical Tumor; cN, clinical Node; cStage, clinical Stage; nCT, neoadjuvant chemotherapy; nICT, neoadjuvant immunochemotherapy; IPTW, inverse probability of treatment weighting; SMD, standardized mean difference; ESS, effective sample size; ECOG PS, Eastern Cooperative Oncology Group Performance Status.

### Pathological response and downstaging

In the unadjusted analysis, the pathological complete response (pCR) rate was 2.50% in the nCT group and 22.32% in the nICT group (*P* < 0.01; **Table 2**). Furthermore, the nICT group showed a more pronounced trend towards downstaging of tumor and lymph node stages compared to the nCT group: T-stage downstaging (48.75% *vs*. 60.27%), N-stage downstaging (42.50% *vs*. 53.11%), and overall TNM stage downstaging (42.50% *vs*. 49.55%), although these differences did not reach statistical significance (all *P* > 0.05; **Table 2**). However, these numerical trends consistently favored the nICT group.

**Table 2:** Surgical and perioperative outcomes between the nCT and nICT groups.

Characteristics	nCT	nICT	P
n	80 (100.00)	224 (100.00)	
Successful R0 resection, n (%)	79 (98.75)	223 (99.55)	0.45
Surgical approach, n (%)			0.11
MIE, n (%)	78 (97.50)	223 (99.55)	
OE, n (%)	2 (2.50)	1 (0.45)	
Pathological response, n (%)			
PCR	2 (2.50)	50 (22.32)	< 0.01
Downstaging of T stage, n (%)	39 (48.75)	135 (60.27)	0.08
Downstaging of N stage, n (%)	34 (42.50)	119 (53.12)	0.12
Downstaging of TNM stage, n (%)	34 (42.50)	111 (49.55)	0.30
Blood loss (mL), median (IQR)	200.00 (200.00–300.00)	200.00 (200.00–300.00)	0.06
Cumulative operative time (min), median (IQR)	261.00 (239.00–310.20)	260.00 (225.00–306.00)	0.43
Number of resected lymph nodes, median (IQR)	18.50 (14.75–26.00)	23.00 (17.00–28.00)	0.02
Number of resected lymph node stations, median (IQR)	8.00 (7.00–9.00)	8.00 (7.00–10.00)	0.46
Chest drainage duration (days), median (IQR)	8.00 (7.00–9.00)	8.00 (7.00–11.00)	0.46
Chest drainage volume (ml), median (IQR)	1310.00 (945.00–1772.50)	1330.00 (830.00–1992.50)	0.75
ICU stay, n (%)	5 (6.25)	31 (13.84)	0.11
ICU stay days, median (IQR)	7.50 (5.0-13.0)	7.00 (6.00-9.00)	0.91
perioperative complications			
Clavien-Dindo grade I-II			
Pulmonary infection, n (%)	6 (7.50)	32 (14.29)	0.17
Cardiac event, n (%)	4 (5.00)	25 (11.16)	0.12
Postoperative hoarseness, n (%)	4 (5.00)	15 (6.70)	0.79
Chylothorax, n (%)	1 (1.25)	7 (3.13)	0.69
Anastomotic leakage, n (%)	6 (7.50)	14 (6.25)	0.90
Clavien-Dindo grade III-IV			
Ascites, n (%)	1 (1.25)	0 (0.00)	0.26
Pleural effusion, n (%)	2 (2.50)	2 (0.89)	0.28
Dysphagia after surgery, n (%)	5 (6.25)	20 (8.93)	0.61
90-day mortality, n (%)	1 (1.25)	8 (3.57)	0.45

MIE, minimally invasive esophagectomy; OE, open esophagectomy; pCR, pathological complete response; TNM, Tumor Node Metastasis.

Notably, achieving pCR was associated with better survival outcomes. In the entire unweighted cohort, patients who achieved pCR had significantly longer OS (HR = 0.43, 95% CI: 0.19–1.00, *P* = 0.04) and DFS (HR = 0.34, 95% CI: 0.18–0.66, *P* < 0.01) compared to patients who did not achieve pCR (**Supplementary Figure 1**).

### Perioperative safety and complications

Patients in both the nCT and nICT groups primarily underwent minimally invasive esophagectomy (MIE), with a small number undergoing open esophagectomy (OE). The R0 resection rates were similar in both groups (99.55% *vs*. 98.75%, *P* = 0.45). There were no significant differences in operative time, blood loss, chest tube drainage volume, or duration of chest tube drainage. However, the number of lymph nodes resected was significantly higher in the nICT group compared to the nCT group (*P* = 0.02; **Table 2**).

Regarding postoperative complications, the incidence of Clavien–Dindo grade I–II complications was slightly higher in the nICT group, but the difference was not statistically significant. Similarly, there were no significant differences in grade III–IV complications, 90-day mortality, or ICU stay duration. The 90-day mortality rate was 3.57% in the nICT group and 1.25% in the nCT group (*P* = 0.45), and 13.8% of patients in the nICT group were admitted to the ICU compared to 6.3% in the nCT group (*P* = 0.11; **Table 2**).

In terms of treatment-related adverse events (TRAEs), the safety profile of nICT was generally manageable and comparable to nCT. Most adverse events were mild (grade 1–2). Although there were numerical differences in hematological toxicities [e.g., anemia (13.40% *vs*. 10.00%, *P* = 0.43) and leukopenia (12.50% *vs*. 17.50%, *P* = 0.36)], these differences were not statistically significant. Similarly, the incidence of grade 3–4 toxicities was very low in both groups. Furthermore, there were no significant differences between the two groups in non-hematological toxicities or treatment delays (3.60% in the nICT group *vs*. 8.75% in the nCT group, *P* = 0.07) (**Supplementary Table 1**).

### Overall survival and disease-free survival analyses

To evaluate long-term efficacy, we analyzed OS and DFS in the original and weighted cohorts. The median follow-up time for survivors was 34.30 months (95% CI: 31.27–38.03) in the nICT group and 46.73 months (95% CI: 41.03–53.07) in the nCT group.

**Figure 2:**
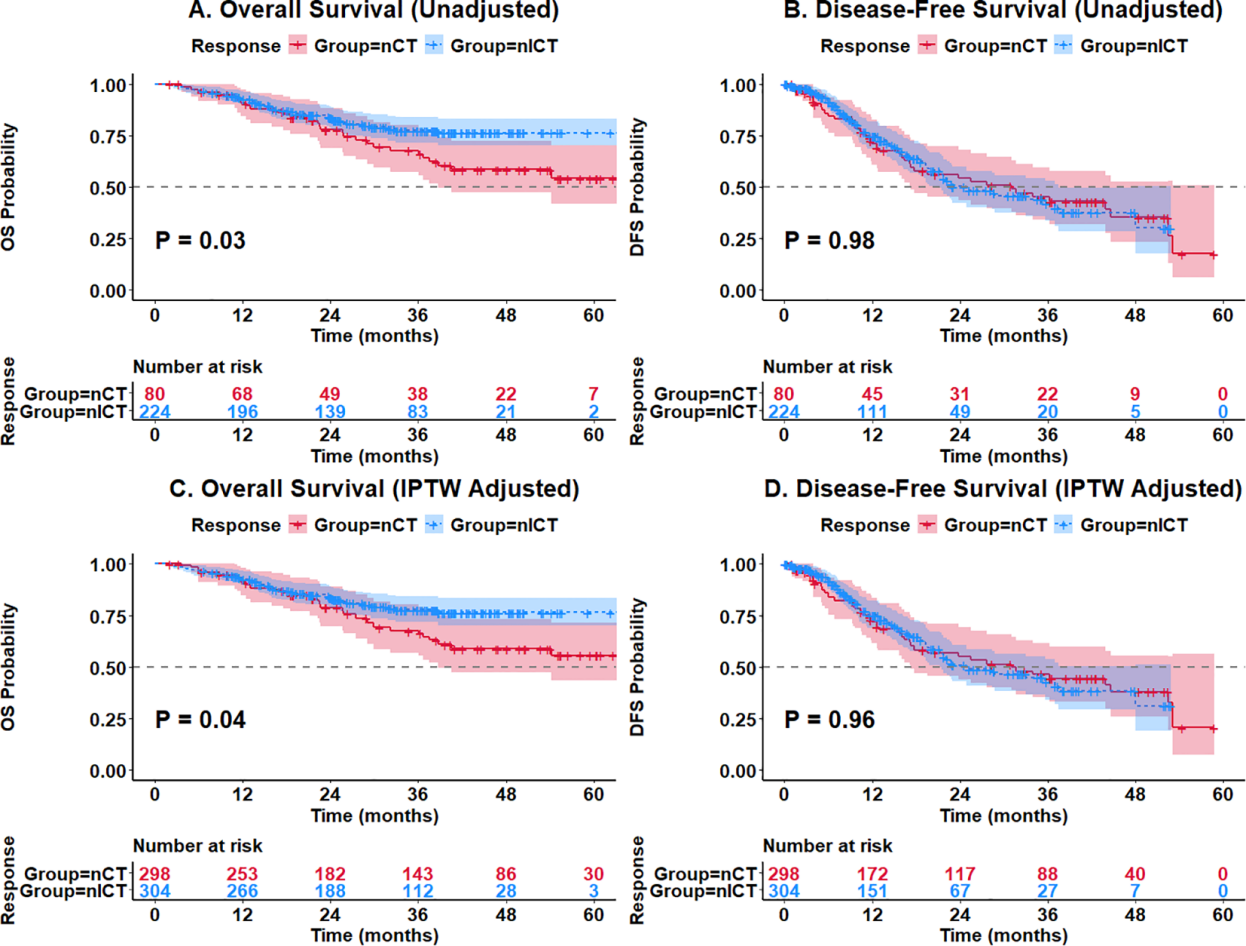
Kaplan–Meier curves for OS and DFS. **(A, B)** Unadjusted analysis for OS and DFS. **(C, D)** IPTW-adjusted analysis for OS and DFS.

In the unadjusted analysis, patients in the nICT group had significantly better overall survival than those in the nCT group (HR = 0.58, 95% CI: 0.36–0.94, *P* = 0.03; **Figure 2A**). However, there was no significant difference in disease-free survival between the two groups (HR = 1.00, 95% CI: 0.68–1.48, *P* = 0.98; **Figure 2B**).

To reduce selection bias, we performed IPTW analysis. In the weighted cohort, the survival benefit remained consistent. The nICT group still maintained a significant overall survival advantage compared to the nCT group (**Figure 2C**). The adjusted 1-year, 2-year, and 3-year overall survival rates for the nICT group were 93.14%, 83.82%, and 77.68%, respectively, while those for the nCT group were 91.13%, 78.81%, and 67.49% (adjusted HR = 0.61, 95% CI: 0.37–0.98, *P* = 0.04). Conversely, consistent with the unadjusted results, there was no statistically significant difference in disease-free survival between the weighted groups (**Figure 2D**), with an adjusted HR of 1.01 (95% CI: 0.67–1.53, *P* = 0.96).

### Recurrence patterns

To investigate the potential reasons for the differences in OS and DFS benefits, we further analyzed the recurrence patterns. Although the overall recurrence rates were similar between the two groups, there were significant differences in the distribution of recurrence sites. Specifically, the distant recurrence rate was significantly higher in the nCT group compared to the nICT group (33.75% *vs*. 18.30%, *P* < 0.01), which was mainly due to an increased incidence of distant organ metastasis (26.25% *vs*. 10.71%, *P* < 0.01). In contrast, no significant differences were observed in regional recurrence (11.25% *vs*. 16.96%, P = 0.22), including local tumor recurrence (7.50% *vs*. 7.14%, *P* = 0.90) and regional lymph node recurrence (3.75% *vs*. 9.82%, *P* = 0.09), or distant lymph node metastasis (7.50% *vs*. 7.59%, *P* = 0.98).

### Univariate and multivariate analysis for OS and DFS

Cox regression analysis showed that nICT independently prolonged overall survival (OS), as confirmed by both univariate analysis (HR = 0.58, 95% CI: 0.36–0.94, *P* = 0.03) and IPTW-adjusted multivariate analysis (HR = 0.61, 95% CI: 0.37–0.98, *P* = 0.04). In the univariate analysis, other baseline clinical variables, including age, sex, smoking status, alcohol consumption, tumor location, clinical stage, cT/cN stages, ECOG PS, or number of neoadjuvant treatment cycles, were not significantly associated with OS. For disease-free survival (DFS), none of the variables, including the treatment group, were significantly associated with prognosis (all *P* > 0.05; **Table 3**).

**Table 3:** OS-related and DFS-related univariate and multivariate analysis.

Variables	OS HR (95% CI), *P* (Univariate)	OS HR (95% CI), *P* (Multivariate) *	DFS HR (95% CI), *P* (Univariate)	DFS HR (95% CI), *P* (Multivariate) *
Treatment				
nCT (Ref)	1.00	1.00	1.00	1.00
nICT	0.58 (0.36–0.94), 0.03	0.61 (0.37–0.98), 0.04	1.00 (0.68–1.48), 0.98	1.01 (0.67–1.53), 0.96
Age	1.01 (0.98–1.04), 0.58	—	1.00 (0.97–1.02), 0.72	—
Sex (Female *vs*. Male)	1.37 (0.63–2.99), 0.43	—	1.00 (0.51–1.97), 1.00	—
Smoking (Yes *vs*. No)	0.94 (0.53–1.69), 0.84	—	0.90 (0.58–1.42), 0.66	—
Alcohol (Yes *vs*. No)	1.37 (0.74–2.55), 0.32	—	1.39 (0.86–2.24), 0.18	—
ECOG PS	0.80 (0.29–2.20), 0.67	—	0.49 (0.18–1.32), 0.16	—
Clinical Stage				
Stage II (Ref)	1.00	—	1.00	—
Stage III	1.27 (0.72–2.25), 0.42	—	1.26 (0.83–1.92), 0.28	—
Stage IV	1.47 (0.33–6.48), 0.61	—	1.58 (0.48–5.24), 0.46	—
cT Stage				
T1-2 (Ref)	1.00	—	1.00	—
T3	0.69 (0.33–1.45), 0.33	—	0.81 (0.43–1.51), 0.51	—
T4	NE	—	2.25 (0.29–17.6), 0.44	—
cN Stage				
N0 (Ref)	1.00	—	1.00	—
N1	1.15 (0.61–2.15), 0.67	—	1.26 (0.79–2.03), 0.33	—
N2	1.04 (0.57–1.92), 0.90	—	1.20 (0.76–1.91), 0.44	—
N3	0.87 (0.11–6.59), 0.89	—	0.54 (0.07–3.99), 0.55	—
Therapy cycles	0.88 (0.53–1.46), 0.63	—	1.10 (0.76–1.58), 0.63	—
Location				
Upper (Ref)	1.00	—	1.00	—
Middle	0.83 (0.29–2.37), 0.73	—	1.02 (0.41–2.58), 0.96	—
Lower	1.03 (0.37–2.90), 0.95	—	1.21 (0.48–3.04), 0.69	—

OS, overall survival; DFS, disease-free survival; HR, hazard ratio; CI, confidence interval; nCT, neoadjuvant chemotherapy; nICT, neoadjuvant immunochemotherapy; ECOG PS, Eastern Cooperative Oncology Group Performance Status; NE, not estimable; Ref, reference group. *Multivariate analysis was performed using inverse probability of treatment weighting (IPTW). The adjusted HR was calculated using a weighted Cox proportional hazards model to account for baseline differences.

### Subgroup analyses of OS and DFS

**Figure 3:**
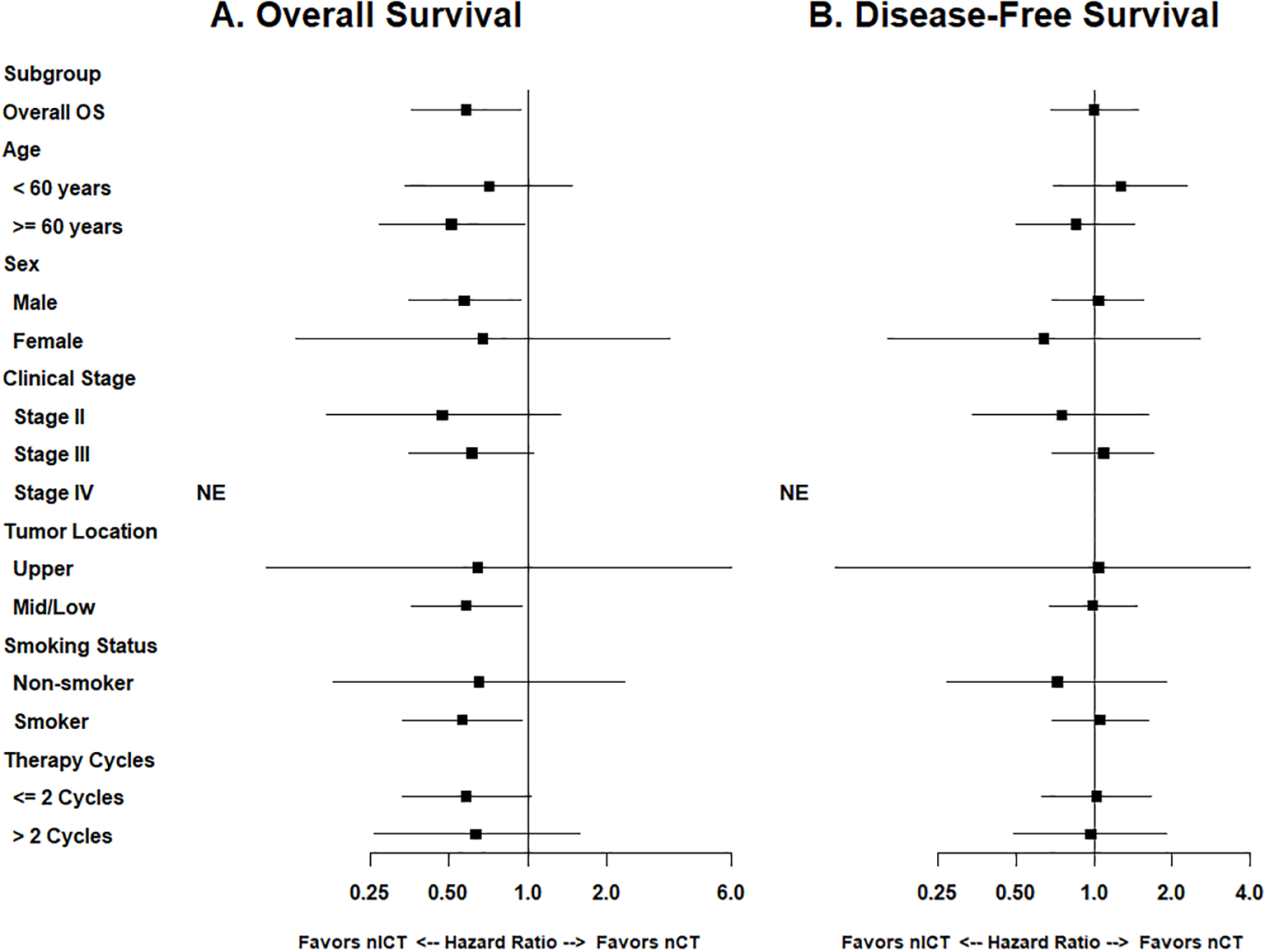
Forest plot of subgroup analysis for overall survival and disease-free survival. **(A)** Overall survival. **(B)** Disease-free survival. Hazard ratios (HRs) and 95% confidence intervals (CIs) were derived from unadjusted Cox proportional hazards models. The vertical solid line indicates an HR of 1.0; values < 1.0 favor the nICT group. NE, not estimable; nICT, neoadjuvant immunochemotherapy; nCT, neoadjuvant chemotherapy.

To further evaluate the efficacy of nICT across different clinical variables, we performed subgroup analyses. In terms of overall OS, nICT demonstrated consistent benefits across most subgroups (**Figure 3A**). Specifically, significant improvements were observed in patients aged ≥ 60 years (*P* = 0.04), male patients (*P* = 0.03), smokers (*P* = 0.03), and patients with tumors located in the middle and lower thoracic segments (*P* = 0.03). Conversely, disease-free survival (DFS) results were similar between the two groups (HR = 1.00, 95% CI: 0.68–1.48, *P* = 0.98; **Figure 3B**). No significant treatment-covariate interactions were detected (all interaction *P* > 0.05). Detailed numerical data from the subgroup analyses are provided in **Supplementary Table 2**.

### mIHC results

**Figure 4:**
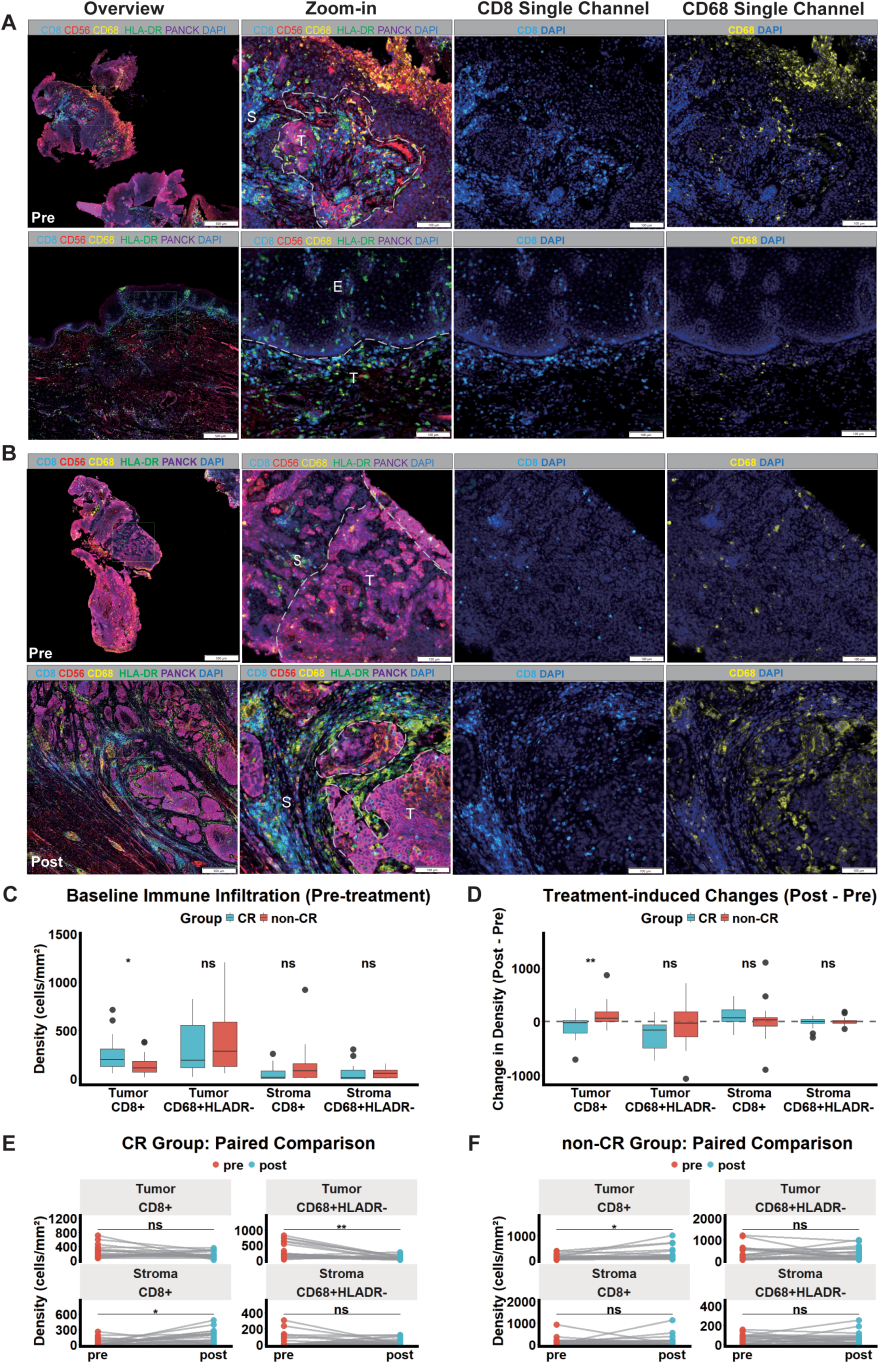
Spatial remodeling of the tumor immune microenvironment induced by neoadjuvant immunochemotherapy. **(A, B)** Representative mIHC images from the CR **(A)** and non-CR **(B)** groups. Top and bottom rows display pre-treatment (baseline) and matched post-treatment samples, respectively. Columns from left to right show the whole-slide overview (scale bar = 500 μm), zoomed-in multispectral regions (scale bar = 100 μm), and single-channel views for CD8/DAPI and CD68/DAPI. Markers: CD8 (cyan), CD56 (red), CD68 (yellow), HLA-DR (green), PANCK (magenta, tumor/epithelium), and DAPI (blue, nuclei). White dashed lines demarcate tumor nests (T), stroma (S), and adjacent normal epithelium (E). **(C)** Baseline infiltration densities of CD8^+^ T cells and CD68^+^HLA-DR^-^ M2-like macrophages in the CR versus non-CR groups. **(D)** Treatment-induced density changes (Δ = Post - Pre) of these immune subsets between the two groups. **(E, F)** Dynamic changes in cell densities within tumor and stromal compartments from baseline to post-treatment in the CR **(E)** and non-CR **(F)** groups. ns, not significant; *P < 0.05, P < 0.01. Abbreviations: CR, complete response.

mIHC analysis revealed distinct immune microenvironment profiles between the two groups. Representative mIHC staining images showcasing the spatial distribution of these immune cells pre- and post-treatment are presented for the CR and non-CR groups in **Figures 4A, B**, respectively.

At baseline, the PD-L1 positivity rate (CPS ≥ 1) was significantly higher in the CR group compared to the non-CR group (65.0% *vs*. 10.0%, *P* < 0.01; **Supplementary Figure 2**). Consistently, the density of intratumoral CD8^+^ T cells was also significantly higher in the CR group than in the non-CR group (*P* = 0.03; **Figure 4C**; **Supplementary Table 3**).

Following treatment, the non-CR group showed a significant increase in CD8^+^ T cells within the tumor nests (P = 0.02; **Figure 4F**; **Supplementary Table 5**), and this density change (Δ) was significantly greater compared to the CR group (P = 0.01; **Figure 4D**; **Supplementary Table 4**). However, M2-like macrophages (CD68^+^HLA-DR^-^) in the non-CR tumors persisted at high levels without a significant reduction (P = 0.56; **Figure 4F**; **Supplementary Table 5**).

Conversely, in the CR group, intratumoral CD8^+^ T cells exhibited a numerical decrease post-treatment, though not statistically significant (*P* = 0.16; **Figure 4E**; **Supplementary Table 5**). Instead, these T cells significantly aggregated within the stromal compartment (*P* < 0.01; **Figure 4E**; **Supplementary Table 5**). Concurrently, a marked reduction in intratumoral M2-like macrophages was observed exclusively in CR patients (*P* < 0.01; **Figure 4E**; **Supplementary Table 5**).

## Discussion

ESCC is a malignancy with a high global disease burden and limited long-term survival, particularly in regions with high incidence such as East Asia (22, 23). The emergence of neoadjuvant immunochemotherapy (nICT) has provided new hope, but high-quality real-world evidence comparing nICT and nCT is still limited (11, 12, 24).

In this real-world cohort study, we demonstrated that nICT significantly improved pCR rates and OS compared with nCT, while DFS showed no significant difference between the two groups. Multivariate analysis further identified nICT as an independent prognostic factor for OS. These findings are consistent with results from previous clinical trials and real-world studies, validating the efficacy of immunotherapy in routine clinical practice.

The survival benefit of nICT primarily stems from its ability to induce deep pathological responses. In our study, patients achieving pCR demonstrated significantly improved outcomes in both OS and DFS (**Supplementary Figure 1**), confirming that eradicating the primary tumor is crucial for long-term survival and highlighting the utility of pCR as a reliable surrogate endpoint (25–27). From a biological perspective, this may reflect a synergistic effect: chemotherapy induces immunogenic cell death and releases antigens (28, 29), thereby “activating” the tumor microenvironment and allowing immune checkpoint inhibitors (ICIs) to reactivate exhausted T cells (30). This combination therapy improves systemic control by eliminating micrometastases, ultimately reducing the rate of distant recurrence (31, 32). These findings directly add to our overall conclusions by confirming that the potent local tumor clearance induced by nICT effectively translates into durable, systemic long-term survival benefits.

Interestingly, at the overall cohort level, although nICT significantly improved OS, disease-free survival (DFS) was similar in both groups (P = 0.96). This discrepancy may be related to changes in recurrence patterns. We found that nICT specifically reduced distant organ metastases, which are the leading cause of death in patients with ESCC (33). Furthermore, this dissociation may also be attributed to the unique kinetics of immunotherapy. Since DFS events typically occur earlier and more frequently, survival curves may initially converge. However, the addition of immunotherapy establishes durable systemic immune memory. Therefore, late-stage immune-mediated survival benefits may emerge after the initial DFS events, creating a “long-tail” effect that ultimately leads to a significant improvement in OS even without a significant difference in DFS. In addition, this sustained immune surveillance may also prolong survival after recurrence, reduce the aggressiveness of recurrence, and ultimately contribute to improved overall survival (34, 35).

In terms of safety, the addition of immunotherapy did not affect the feasibility of surgery. We found no significant differences in surgical time, blood loss, and postoperative recovery between the two groups of patients. Notably, the nICT group had a significantly higher number of lymph nodes removed. This may reflect easier tissue dissection after tumor regression, thus facilitating more thorough lymph node dissection. Postoperative complications and 90-day mortality were similar. Although the nICT group experienced slightly more mild hematological toxicities, these were all clinically manageable. Overall, these results confirm that nICT is a safe and well-tolerated neoadjuvant treatment regimen.

Our multiplex immunohistochemistry analysis revealed distinct spatial patterns of immune dynamics. Consistent with previous reports, a high density of CD8^+^ T cells at baseline predicted pCR, confirming the importance of a “hot” tumor microenvironment (36, 37). However, a key difference emerged after treatment. Although CD8^+^ T cells significantly increased in non-pCR tumors, this did not improve prognosis, possibly due to persistent M2-like macrophage-mediated suppression. In contrast, the pCR group showed a significant reduction in M2-like macrophages, accompanied by an accumulation of stromal CD8^+^ T cells, which is highly consistent with the pathological clearance of the tumor bed; as tumor nests are eradicated by nICT, the original “target zone” disappears, leading to T cell retention in the surrounding stroma. These findings suggest that eliminating macrophage-mediated immunosuppression, rather than merely expanding T cell density, is crucial for the efficacy of nICT (38, 39). Additionally, elevated baseline PD-L1 levels in responders indicate a favorable immune context for treatment efficacy (40). Moreover, recent studies have highlighted the significant potential of chemotherapy combined with immunotherapy and local treatment in LA-ESCC. However, as Ma and Baran recently pointed out, the immunosuppressive tumor microenvironment remains a key obstacle to achieving optimal therapeutic outcomes. Our mIHC results directly support this view, indicating that the persistent presence of M2-like macrophages restricts CD8^^+^^ T cell spatial distribution and anti-tumor function, which may explain the observed differential pathological responses and survival outcomes (41).

This study has several limitations. First, the retrospective, single-center design limits the general applicability of the results, although we employed IPTW to minimize bias. Second, the small sample size and significant asymmetry between the nICT and nCT groups not only limit the statistical power of our DFS and subgroup analyses but also pose challenges to our IPTW model. Specifically, this imbalance may lead to unstable weights and variance inflation. Third, our mIHC analyses are exploratory and were conducted exclusively within the nICT cohort. Because direct comparative TME profiling with the nCT cohort was not performed, we cannot conclusively attribute the observed immune dynamics solely to immune checkpoint inhibition. Additionally, while we used the CD68^+^HLA-DR^-^ signature to define M2-like macrophages, specific markers such as CD163 were lacking. Therefore, these response-associated spatial dynamics require further validation in prospective studies with parallel nCT cohorts and complementary functional assays. Finally, differences in patient treatment regimens may introduce confounding factors. Nevertheless, the real-world evidence provided by this study can complement controlled clinical trials.

In conclusion, this real-world study demonstrates that neoadjuvant immunochemotherapy improves pathological response and overall survival in resectable LA-ESCC compared with chemotherapy alone. The mIHC findings suggest that enhanced CD8^+^ T-cell activity and reduced M2-like macrophages may underlie this benefit. Future prospective studies with biomarker analyses are warranted to validate these results and optimize patient selection for nICT.

## Data Availability

The raw data supporting the conclusions of this article will be made available by the authors, without undue reservation.
