# Supplementary material for: Survival benefit and dynamics of CD8+ T cells and tumor-associated macrophages in neoadjuvant immunochemotherapy vs. chemotherapy for locally advanced esophageal squamous cell carcinoma: an IPTW-adjusted real-world study
Source: Front Immunol. 2026 Mar 26;17:1788756. doi: 10.3389/fimmu.2026.1788756 (PMC13061859; doi:10.3389/fimmu.2026.1788756)
Supplement: Supplementary file 1 [file Table1.docx]

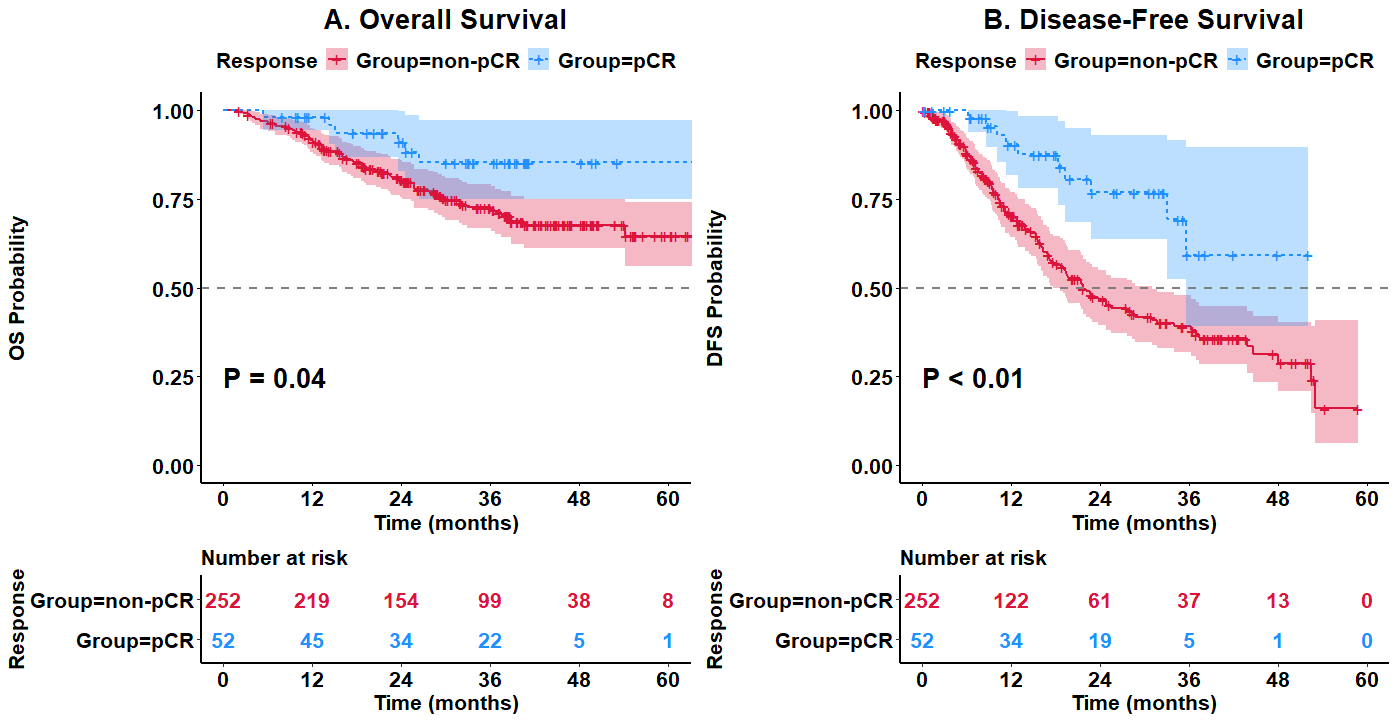


Supplementary Figure 1 Kaplan-Meier survival curves comparing patients with and without pathological complete response (pCR) in the unweighted cohort.


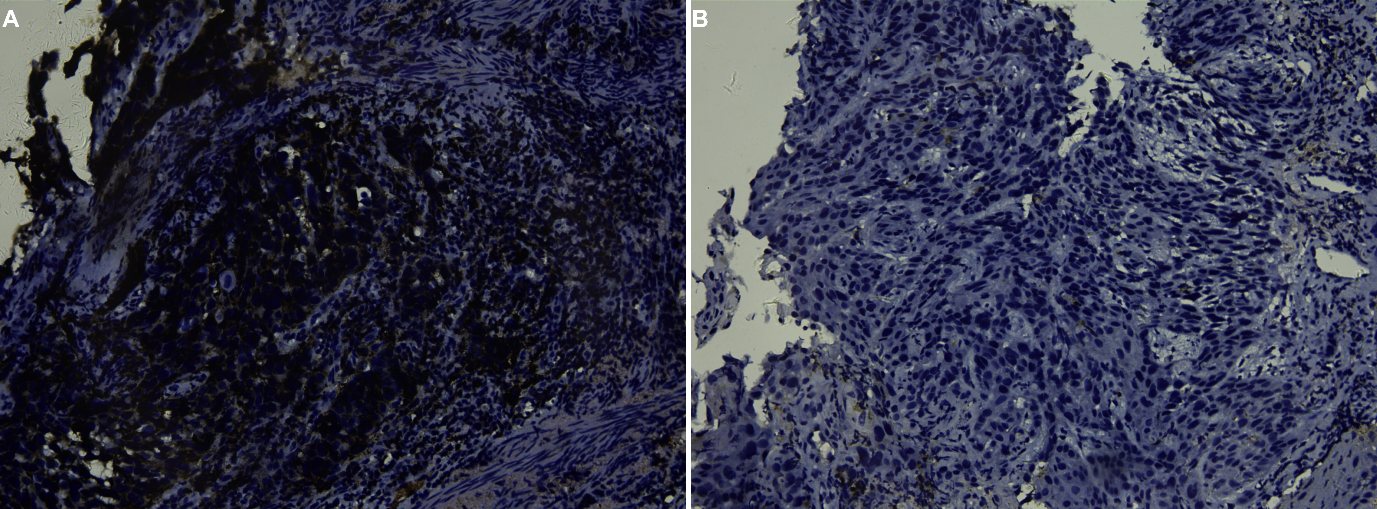


Supplementary Figure 2 Representative baseline PD-L1 immunohistochemistry (IHC) images. (A) Positive PD-L1 expression (Combined Positive Score [CPS] ≥ 1), frequently observed in the CR group. (B) Negative PD-L1 expression (CPS < 1), predominantly observed in the non-CR group. Scale bar = 100 μm.

Supplementary Table 1. Treatment-related adverse events between the nCT and nICT groups.

| Adverse event | nCT,n (%)  Grade 1-2 | nICT,n (%)  Grade 1-2 | P | nCT,n (%)  Grade 3-4 | nICT, n (%)  Grade 3-4 | P |
| --- | --- | --- | --- | --- | --- | --- |
| Hematologic toxicities | |  |  |  |  |  |
| Anemia | 8 (10.00) | 30 (13.40) | 0.43 | 0 (0.0) | 1 (0.45) | – |
| Leukopenia | 14 (17.50) | 28 (12.50) | 0.36 | 0 (0.0) | 1 (0.45) | – |
| Neutropenia | 5 (6.25) | 24 (10.70) | 0.32 | 0 (0.0) | 0 (0.0) | – |
| Thrombocytopenia | 2 (2.50) | 4 (1.80) | – | 0 (0.0) | 2 (0.9) | – |
| Non-hematologic toxicities | |  |  |  |  |  |
| AST increased | 9 (11.30) | 13 (5.80) | 0.15 | 0 (0.0) | 1 (0.45) | – |
| ALT increased | 7 (8.75) | 24 (10.70) | 0.78 | 0 (0.0) | 2 (0.9) | – |
| Hyperbilirubinemia | 0 (0.00) | 5 (2.20) | 0.56 | 0 (0.0) | 1 (0.45) | – |
| Rash | 1 (1.25) | 2 (0.90) | – | 0 (0.0) | 0 (0.0) | – |
| Vomiting | 1 (1.25) | 0 (0.00) | – | 0 (0.0) | 0 (0.0) | – |
| Nausea | 2 (2.50) | 1 (0.45) | 0.17 | 0 (0.0) | 0 (0.0) | – |
| Asthenia | 1 (1.25) | 1 (0.45) | 0.46 | 0 (0.0) | 0 (0.0) | – |
| Treatment delay | 7 (8.75) | 8 (3.60) | 0.07 | – | – | – |
| Surgical delay | 1 (1.25) | 4 (1.80) | – | – | – | – |

nCT, neoadjuvant chemotherapy; nICT, neoadjuvant immunochemotherapy; AST, aspartate aminotransferase; ALT, alanine aminotransferase; “–” indicates no statistical analysis was performed due to low event numbers.

Supplementary Table S2. Subgroup analysis of overall survival and disease-free survival.

| Subgroup | OS HR (95% CI) | P value | P for interaction | DFS HR (95% CI) | P value | P for interaction |
| --- | --- | --- | --- | --- | --- | --- |
| Overall Cohort | 0.58 (0.36–0.94) | 0.03 | — | 1.00 (0.68–1.48) | 0.98 | — |
| Age |  |  | 0.46 |  |  | 0.23 |
| < 60 years | 0.71 (0.34–1.47) | 0.36 |  | 1.27 (0.70–2.30) | 0.44 |  |
| ≥ 60 years | 0.51 (0.27–0.97) | 0.04 |  | 0.85 (0.50–1.44) | 0.54 |  |
| Sex |  |  | 0.83 |  |  | 0.56 |
| Male | 0.57 (0.35–0.94) | 0.03 |  | 1.04 (0.69–1.55) | 0.87 |  |
| Female | 0.67 (0.13–3.46) | 0.63 |  | 0.64 (0.16–2.57) | 0.53 |  |
| Clinical Stage |  |  | 0.53 |  |  | 0.21 |
| Stage II | 0.47 (0.17–1.33) | 0.16 |  | 0.75 (0.34–1.62) | 0.46 |  |
| Stage III | 0.61 (0.35–1.05) | 0.07 |  | 1.09 (0.69–1.71) | 0.72 |  |
| Stage IV | NE | NE |  | NE | NE |  |
| Tumor Location |  |  | 0.97 |  |  | 0.91 |
| Upper | 0.64 (0.07–6.15) | 0.70 |  | 1.04 (0.10–10.46) | 0.98 |  |
| Middle / Lower | 0.58 (0.36–0.95) | 0.03 |  | 0.99 (0.67–1.47) | 0.97 |  |
| Smoking Status |  |  | 0.76 |  |  | 0.33 |
| Non-smoker | 0.65 (0.18–2.34) | 0.51 |  | 0.72 (0.27–1.91) | 0.52 |  |
| Smoker | 0.56 (0.33–0.95) | 0.03 |  | 1.06 (0.69–1.63) | 0.78 |  |
| Therapy Cycles |  |  | 0.91 |  |  | 0.89 |
| ≤ 2 Cycles | 0.58 (0.33–1.02) | 0.06 |  | 1.02 (0.63–1.66) | 0.93 |  |
| > 2 Cycles | 0.63 (0.26–1.57) | 0.33 |  | 0.97 (0.49–1.92) | 0.93 |  |

OS, overall survival; DFS, disease-free survival; HR, hazard ratio; CI, confidence interval; NE, not estimable.

Supplementary Table S3. Baseline immune cell densities in CR and non-CR patients before treatment (mIHC analysis).

| Marker | CR (Mean ± SD) | non-CR (Mean ± SD) | P value |
| --- | --- | --- | --- |
| Tumor: CD56(dim)+ cells | 310.11 ± 525.72 | 601.73 ± 768.41 | 0.19 |
| Tumor: CD56(bright)+ cells | 104.69 ± 281.28 | 87.42 ± 154.83 | 0.77 |
| Tumor: CD8+ cells | 254.31 ± 209.79 | 136.58 ± 95.31 | 0.03 |
| Tumor: CD68+HLA-DR+ cells | 273.52 ± 276.27 | 249.41 ± 205.88 | 0.71 |
| Tumor: CD68+HLA-DR− cells | 307.88 ± 289.38 | 361.62 ± 335.63 | 0.52 |
| Stroma: CD56(dim)+ cells | 64.49 ± 164.76 | 229.22 ± 475.94 | 0.12 |
| Stroma: CD56(bright)+ cells | 16.22 ± 33.22 | 50.48 ± 122.63 | 0.20 |
| Stroma: CD8+ cells | 67.10 ± 99.86 | 90.54 ± 178.93 | 0.52 |
| Stroma: CD68+HLA-DR+ cells | 42.54 ± 74.50 | 62.72 ± 80.77 | 0.37 |
| Stroma: CD68+HLA-DR− cells | 33.29 ± 67.77 | 60.02 ± 99.47 | 0.28 |

Abbreviations: CR, complete response; non-CR, non-complete response; SD, standard deviation. Values are presented as mean ± SD.

Supplementary Table S4. Between-group comparison of Δ (Post–Pre) immune cell density changes in CR and non-CR patients.

| Marker | CR (Mean ± SD) | non-CR (Mean ± SD) | P value |
| --- | --- | --- | --- |
| Tumor: CD56(dim)+ cells | −28.65 ± 405.02 | 182.68 ± 540.23 | 0.32 |
| Tumor: CD56(bright)+ cells | −19.06 ± 185.06 | −14.44 ± 73.26 | 0.91 |
| Tumor: CD8+ cells | −79.59 ± 223.37 | 124.96 ± 232.76 | 0.01 |
| Tumor: CD68+HLA-DR+ cells | −16.57 ± 379.61 | 54.68 ± 471.11 | 0.57 |
| Tumor: CD68+HLA-DR− cells | −228.24 ± 282.36 | −52.27 ± 410.54 | 0.13 |
| Stroma: CD56(dim)+ cells | 42.63 ± 328.19 | 21.79 ± 427.66 | 0.79 |
| Stroma: CD56(bright)+ cells | 9.93 ± 80.60 | −4.40 ± 128.13 | 0.74 |
| Stroma: CD8+ cells | 101.28 ± 174.02 | 34.24 ± 361.67 | 0.46 |
| Stroma: CD68+HLA-DR+ cells | 22.18 ± 184.47 | −10.62 ± 223.06 | 0.70 |
| Stroma: CD68+HLA-DR− cells | −44.19 ± 199.71 | 40.21 ± 253.19 | 0.28 |

Abbreviations: CR, complete response; non-CR, non-complete response; Δ, post-treatment minus pre-treatment; SD, standard deviation. Values are presented as mean ± SD.

Supplementary Table S5. Within-group comparison of pre- and post-treatment immune cell densities in CR and non-CR patients.

| Marker | Group | Pre-treatment (Mean ± SD) | Post-treatment (Mean ± SD) | P value |
| --- | --- | --- | --- | --- |
| Tumor: CD56(dim)+ cells | CR | 310.11 ± 525.72 | 281.46 ± 398.47 | 0.78 |
|  | non-CR | 601.73 ± 768.41 | 784.41 ± 1000.74 | 0.42 |
| Tumor: CD56(bright)+ cells | CR | 104.69 ± 281.28 | 85.63 ± 246.42 | 0.72 |
|  | non-CR | 87.42 ± 154.83 | 73.06 ± 169.22 | 0.59 |
| Tumor: CD8+ cells | CR | 254.31 ± 209.79 | 174.73 ± 226.55 | 0.16 |
|  | non-CR | 136.58 ± 95.31 | 261.55 ± 261.70 | 0.02 |
| Tumor: CD68+HLA-DR+ cells | CR | 273.52 ± 276.27 | 256.95 ± 340.37 | 0.82 |
|  | non-CR | 249.41 ± 205.88 | 304.09 ± 312.75 | 0.35 |
| Tumor: CD68+HLA-DR− cells | CR | 307.88 ± 289.38 | 79.63 ± 211.24 | <0.01 |
|  | non-CR | 361.62 ± 335.63 | 309.35 ± 397.59 | 0.56 |
| Stroma: CD56(dim)+ cells | CR | 64.49 ± 164.76 | 107.13 ± 355.18 | 0.53 |
|  | non-CR | 229.22 ± 475.94 | 251.01 ± 740.32 | 0.86 |
| Stroma: CD56(bright)+ cells | CR | 16.22 ± 33.22 | 26.15 ± 98.23 | 0.63 |
|  | non-CR | 50.48 ± 122.63 | 46.08 ± 108.54 | 0.90 |
| Stroma: CD8+ cells | CR | 67.10 ± 99.86 | 168.38 ± 201.27 | <0.01 |
|  | non-CR | 90.54 ± 178.93 | 124.78 ± 321.55 | 0.45 |
| Stroma: CD68+HLA-DR+ cells | CR | 42.54 ± 74.50 | 64.73 ± 156.45 | 0.47 |
|  | non-CR | 62.72 ± 80.77 | 52.10 ± 201.40 | 0.70 |
| Stroma: CD68+HLA-DR− cells | CR | 33.29 ± 67.77 | 20.03 ± 107.17 | 0.57 |
|  | non-CR | 60.02 ± 99.47 | 100.23 ± 224.47 | 0.32 |

Abbreviations: CR, complete response; SD, standard deviation. Values are presented as mean ± SD.
